# A Glimpse Into the Microbiome of Sjögren’s Syndrome

**DOI:** 10.3389/fimmu.2022.918619

**Published:** 2022-07-14

**Authors:** Chuiwen Deng, Qiufeng Xiao, Yunyun Fei

**Affiliations:** Department of Rheumatology and Clinical Immunology, National Clinical Research Center for Dermatologic and Immunologic Diseases (NCRC-DID), Ministry of Science and Technology, State Key Laboratory of Complex Severe and Rare Diseases, Peking Union Medical College Hospital, Key Laboratory of Rheumatology and Clinical Immunology, Ministry of Education, Chinese Academy of Medical Sciences and Peking Union Medical College, Beijing, China

**Keywords:** microbiome, autoimmunity, inflammation, Sjogren’s syndrome, pathogenesis

## Abstract

Sjögren’s syndrome (SS) is a common chronic systemic autoimmune disease and its main characteristic is lymphoid infiltration of the exocrine glands, particularly the salivary and lacrimal glands, leading to sicca symptoms of the mouth and eyes. Growing evidence has shown that SS is also characterized by microbial perturbations like other autoimmune diseases. Significant alterations in diversity, composition, and function of the microbiota were observed in SS. The dysbiosis of the microbiome correlates with worse symptoms and higher disease severity, suggesting that dysbiosis may be of great importance in the pathogenesis of SS. In this review, we provide a general view of recent studies describing the microbiota alterations of SS, the possible pathways that may cause microbiota dysbiosis to trigger SS, and the existence of the gut-ocular/gut-oral axis in SS.

## Introduction

Sjögren’s Syndrome (SS) is a chronic autoimmune disease with a female-to-male predominance of 9:1, of which the peak incidence is about 50 years of age (1). SS primarily causes inflammation of the exocrine glands (predominantly the salivary and lacrimal glands), resulting in severe oral and ocular dryness. The clinical manifestation of SS is heterogeneous, which can differ from sicca symptoms to systemic disease and lymphoma (2). The significant immunologic abnormalities of SS, including the presence of serum anti-SSA antibodies and focal lymphocytic sialadenitis on biopsy of labial salivary glands, is the basis of formal criteria for the diagnosis (3). To date, little is known about the pathogenesis of SS. A wide range of environmental factors interacting with the genetic predisposition of an individual may affect the development of SS.

The Human Microbiome Project has revealed that the microbiome plays an outstanding role in the pathogenesis of autoimmune diseases (4). Numerous studies of human autoimmune disease consistently report a state of microbial dysbiosis. For instance, dysbiosis of gut microbiota has been demonstrated as a characteristic of some rheumatic diseases including, rheumatoid arthritis (RA), systemic lupus erythematosus (SLE), and systemic sclerosis (5–9). Recently, many studies have described the alterations due to SS of microbial communities and the composition of the intestinal, oral, and ocular areas, though some results were heterogeneous. Furthermore, dysbiosis is associated with the worsening of symptoms and disease activity in SS patients. However, the cause or effect role that microbiome plays in the pathogenesis of SS remains unclear.

This review aims to describe the microbiome alterations of SS, the clinical association of microbiome dysbiosis, and substantial mechanisms of the microbiota involved in the pathology of SS.

## Diversity changes in microbiota

Microbiota diversity is essential to maintain the stability and efficiency of the ecosystem. The ratio of *Firmicutes* to *Bacteroidetes* (F/B ratio) has a close connection with the composition of the intestinal microbiota, which is frequently used to evaluate the imbalance of intestinal microbiota (7, 10). The F/B ratio has been found to be lower than the normal level in several diseases, including inflammatory bowel disease, RA, SLE, and obesity (11). Interestingly, recent studies demonstrated that a lower F/B ratio in SS patients was a main characteristic of the gut microbiota composition compared with HCs, which means *Firmicutes* were suppressed and *Bacteroidetes* became conditional pathogens and also indicate the existence of gut microbial dysbiosis in SS (12–14). In addition, significantly decreased diversity of SS was found when compared with healthy controls (15–21), implying that microbiota dysbiosis may participate in the pathogenesis. However, the mechanism of microbiome alteration in SS was still known.

Both the specific symptoms (dry mouth and dry eye) and autoimmunity (the specific disease background of rheumatoid disease) are potential factors that may influence the constitution of the microbiome. Between SS and symptom controls, most of the studies observed no significant difference in the gut, oral, and ocular microbiota (13, 22–25), which indicates that the changes in the microbiome might be only associated with dry eye or dry mouth (**Table 1**).

**Table 1:** Microbiome studies of Sjögren’s syndrome.

Ref.	Year	Sample	Methods	Case and control	Diversity changes in SS compared with controls				
					Diversity	Phylum		Genus	
						Increased	Decreased	Increased	Decreased
(26)	2016	swabs on bilateral buccal mucosa	16S rRNA gene sequencing of V1-V3 regions	pSS patients, HCs	Not described		*Proteobacteria*	*Leucobacter, Delftia, Pseudochrobactrum, Ralstonia, Mitsuaria*	*Haemophilus, Neisseria, Comamona, Granulicatella, Limnohabitans*
(17)	2016	stool samples	16S rRNA gene sequencing of the V4 region	pSS patients, Human Microbiome Project (HMP) data	No significant changes or differences			*Pseudobutyrivibrio, Escherichia/Shigella, Blautia, Streptococcus, Bifidobacterium, Anaerostipes, Bilophila*	*Bacteroides, Parabacteroides, Faecalibacterium, Odoribacter, Haemophilus, Prevotella*
	2016	tongue samples	16S rRNA gene sequencing of the V4 region	pSS patients,HCs	Decreased Shannon diversity index score			*Streptococcus*	*Leptotrichia, Fusobacterium, Bergeyella, Peptococcus, Butyrivibrio*
	2016	inferior conjunctiva samples	16S rRNA gene sequencing of V1-V3 regions	pSS patients,HCs	No significant changes or differences				
(18)	2016	whole unstimulated saliva	16S rRNA gene sequencing of V1-V2 regions	pSS patients, HCs	Lower alpha diversity	*Firmicutes*	*Synergistetes, Spirochaetes*	*Streptococcus*	*Treponema, Peptostreptococcaceae_[XIII][G-1], Bacteroidaceae_[G-1], Moryella, Fretibacterium, Porphyromonas, Tannerella, Catonella *
(16)	2017	stool samples	16S rRNA gene sequencing	pSS patients,HCs	Not described				*Alistipes, Bifidobacterium*
(22)	2018	buccal swab samples	16S rRNA gene sequencing of the V4 region	pSS patients, non-SS sicca, HCs	No significant changes or differences	Firmicutes/Proteobacteria ratio		*Gemella*	*Streptococcus*
(19)	2018	oral washings	16S rRNA gene sequencing of V3-V4 regions	pSS patients, HCs	Lower alpha diversity but no significant changes in beta diversity			*Veillonella*	*Actinomyces, Haemophilus, Neisseria, Rothia, Porphyromonas, Peptostreptococcus*
(27)	2018	oral washings	16S rRNA gene sequencing of V4-V5 regions	pSS patients, HCs	No significant changes or differences	*Bacteroidetes, Firmicutes*	*Proteobacteria*	*Prevotella, Actinomyces, Peptostreptococcus*	*Neisseria, Streptococcus*
(23)	2018	oral washings	16S rRNA gene sequencing of the V4 region	pSS patients, non-SS sicca, HCs	No significant changes or differences			*Fusobacterium, Selenomonas, Shuttleworthia*	*Haemophilus, Streptococcus, Abiotrophia*
(20)	2019	stimulated whole saliva	16S rRNA gene sequencing of V3-V5 regions	pSS patients, non-SS sicca, HCs	No significant changes or differences			*Veillonella*	*Streptococcus, Haemophilus, Neisseria, Porphyromonas*
(24)	2019	stimulated whole saliva	16S rRNA gene sequencing of V1-V3 regions	pSS patients, non-SS sicca, HCs	No significant changes or differences				
(12)	2019	stool samples	16S rRNA gene sequencing of the V4 region	pSS and SLE patients; population control	No significant changes or differences	*Bacteroidetes, Proteobacteria*	Lower Firmicutes/Bacteroides ratio	*Bacteroides*	
	2019	buccal swab and oral washing	16S rRNA gene sequencing of the V4 region	pSS and SLE patients; population control	Lower richness and diversity	*Firmicutes*	*Proteobacteria*	*Lactobacillus*	*Haemophilus, Neisseria, Granulicatella*
(28)	2020	stimulated saliva	16S rRNA gene sequencing of V3-V4 regions	Nfkbiz-/-mice, Nfkbiz+/+mice	Increased species evenness and richness in Nfkbiz-/-mice	*Actinobacteria, Proteobacteria*	*Firmicutes*		
(15)	2020	stool samples	16S rRNA gene sequencing of V2–4–8 and V3–6, 7–9 regions	pSS patients, HCs	Decreased diversity and richness	*Bacteroidetes, Proteobacteria*	*Firmicutes, Actinobacteria*	*Prevotella, Clostridium, Enterobacter, Escherichia, Veillonella, Streptococcus*	*Bacteroides, Parabacteroides,Faecalibacterium, Roseburia, Ruminococcus, Dorea, Alistipes, Blautia,Lachnospira, Bifidobacterium*
(25)	2020	oral washings	16S rRNA gene sequencing of V1-V3 regions	pSS patients, control subjects (14 without oral dryness and 11 with dryness)	No significant changes or differences	*Firmicutes*	*Proteobacteria, Fusobacteria, TM7, Spirochaetes*	*Streptococcus, Prevotella, Lactobacillus, Atopobium, Staphylococcus*	*Haemophilus, Leptotrichia, Fusobacterium, Lautropia, Neisseria*
(13)	2020	stool samples	16S rRNA gene sequencing of V3-V4 regions	pSS patients, environmental dry eye syndrome (DES), HCs	No significant changes or differences	*Bacteriodetes*	*Actinobacteria*, Firmicutes/Bacteroidete ratio	*Prevotella, Odoribacter, Alistipes*	*Family Clostridia, Bifidobacterium, Blautia, Dorea, Agathobacter*
(21)	2020	Unstimulated saliva by spitting method	16S rRNA gene sequencing of V3-V4 regions	pSS patients, HCs	No significant changes or differences			*Bifidobacterium, Dialister, Lactobacillus*	*Leptotrichia*
(29)	2021	Unstimulated whole saliva	16S rRNA gene sequencing of V3-V4 regions	pSS patients, HCs	No significant changes or differences			*Megasphaera*	*Haemophilus, Aggregatibacter, Abiotrophia, Cardiobacterium, Johnsonella, Bifidobacterium*
(14)	2022	Conjunctival sac swab samples	16S rRNA gene sequencing of V3-V4 regions	pSS patients, non-SS sicca, HCs	Lower alpha diversity (SS and non-SS sicca vs HCs); No significant difference(SS vs non-SS sicca)	*Actinobacteriota*, Firmicutes/Bacteroidetes ratio, *Proteobacteria*	*Bacteroidota*	*Acinetobacter,Corynebacterium, Clostridium_sensu_stricto_1, Geobacillus*	*Bacillus*

Since the correlation between the common clinical symptom and autoimmunity is almost definite, it seems that this specific disease background of SS should also be closely associated with microbiome alteration. However, no difference was found in the richness of gut microbiota but significantly lower diversity of oral microbiota was observed in SS than in SLE (12), which was counted in dry mouth by the researchers (**Table 1**). Along with these reports that questioned the role autoimmunity plays in the microbiota dysbiosis of SS, one possible reason to explain the microbiota changes is that the alteration of the microbiome is the leading factor of clinical symptoms as well as the breakdown of autoimmune system. After all, other studies also reported that microbial dysbiosis can occur independent of salivary deficiency and SS can be developed independent of microbiome changes (18, 30).

Many factors were found to be related to microbiome changes. For example, the oral microbiota composition could be affected by oral conditions such as periodontitis, caries, and age (31, 32). However, the triggers that result in altered microbiota in SS have not yet been clarified.

Because the standard of normal microbiome has not been established, we could not make a definite conclusion on the point that excluding pSS patients *via* characteristics of microbiome is correct. Considering that the pathogenesis of SS is complex, it is possible that not all the patients exert microbiota alteration, which is also observed in some studies (19, 26, 27).

## Correlation between altered microbiota and SS

Microbial diversity has been proven to change in SS as described above. The nature of these changes is one of the most important questions to be answered.

Growing evidence of microbiota dysbiosis in SS confirmed the trend that lower diversity could lead to higher disease activity. An early study reported that oral antibiotic treatment and desiccating stress leads to great alterations in the gut microbiota (17). In addition, the diversity of fecal microbiota was negatively associated with combined ocular and systemic disease index. Similarly, SS patients with severe dysbiosis had a higher ESSDAI total score, ClinESSDAI total score, and levels of fecal calprotectin, while having lower levels of complement component 4 (16). Furthermore, a strong positive relation was found between tear break-up time and both *Actinobacteria* and *Bifidobacteria* (13) (**Table 1**).

To further understand the mechanisms related to these observed correlations, researchers also studied microscopic evidence. In SS, the levels of proinflammation factors IL-6, IL-12, IL-17, and TNF-α were positively associated with the richness of *Enterobacter*, while negatively associated with the abundance of *Lachnospira*, *Roseburia*, *Bifidobacterium*, *Ruminococcus*, *Blautia*, and *Roseburia*. Compared with SS, a significant inverse correlation was only observed between the abundance of *Parabacteroides distasonis* and the levels of IL-6 and TNF-α in controls (15) (**Table 1**). It is intuitive that the germs involved in inflammation were more prevalent in SS and the bacterial communities that participated in the immune regulation were totally different between SS and the controls.

The research noted above made an impression that the microbial alteration of SS has a close association with severe clinical symptoms *via* some inflammation pathways. Regarding the link between microbiota dysbiosis and inflammation pathways, little is known as research regarding the cause in SS is limited. Based on present studies, we summarize three potential mechanisms: molecular mimicry, metabolite changes, and epithelial tolerance breakdown.

## From correlation to potential causality

**Figure 1:**
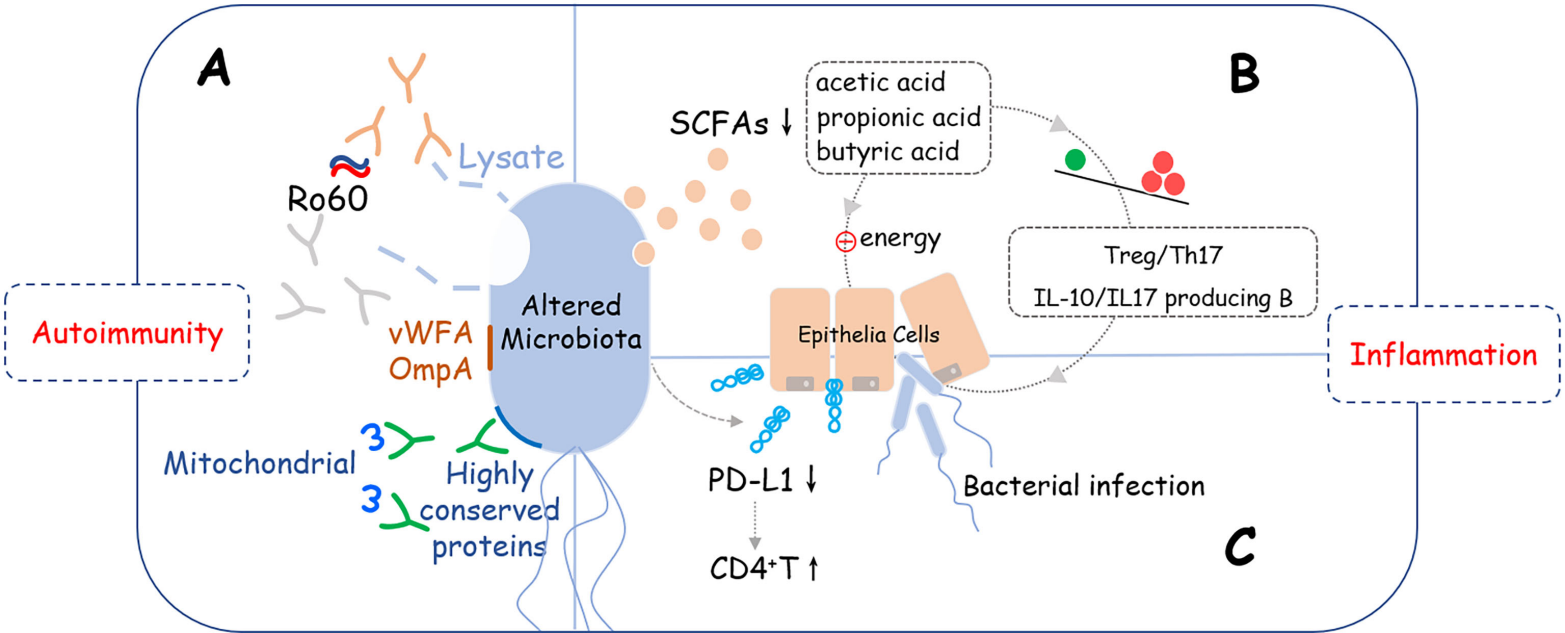
Potential crosslinks between microbiome alteration and pathogenesis of SS. Altered microbiome may induce development of SS *via* three potential pathways: **(A)** Molecular mimicry: Lysates, proteins, or peptides that originate from bacteria, including von Willebrand factor type A domain protein (vWFA), OmpA, and some other highly conserved proteins could react with anti-Ro60 antibodies or SSA/Ro60-reactive T cells, which are involved in the activation of excessive autoimmune response; **(B)** Metabolite changes: Short-chain fatty acids (SCFAs) produced by bacteria were decreased that led to reducing the immune checkpoint molecules and imbalance of T cells (Treg/Th17) and B cells (IL-10/IL-17 producing B cells), which provides a pro-inflammation microenvironment is SS. **(C)** Epithelial tolerance breakdown: Reducing the supply of energy for epithelial cells, disrupting the immunomodulatory function such as down-regulating the expression level of PD-L1 on epithelial cells, and interfering with the mucus barrier are possible mechanisms that epithelial tolerance was broken down.

The microbiome alteration has a negative effect on the pathogenesis of SS, but the causality between them remained unknown. Based on the diversity and correlations described above, we presume that an unknown variant leads to microbiota changes which directly affects the function of organs, for example, salivation. Also, an altered microbiome further activates the immune system which results in the damage of a specific site *via* inflammation. Three pathways might help explain these presumptions (**Figure 1**).

### Molecular Mimicry

Molecular mimicry has been widely acknowledged as a mechanism of autoimmune diseases caused by infectious agents with epitope intermolecular spreading (33, 34). B cell cross-reactivity between Coxsackie virus 2B protein and Ro60 has been reported to have significant association with the initiation and perpetuation of anti-Ro60 autoantibody response, which is essential in the pathology of SS (35). Recently, Greiling et al. found that the serum of anti-Ro60-positive patients can react with the lysates of *Bacteroides thetaiotaomicron* (36). Also, Yanagisawa et al. found a new antigen named OmpA from *E. coli*, which is highly antigenic to elicit antibodies against SSA/Ro and SSB/La and cause inflammation of the Harderian and the salivary glands (37), suggesting that microbe-originated molecules elicit inflammation in exocrine glands with specificity.

Interestingly, Szymula et al. also identified a peptide originating from *C. ochracea*, named von Willebrand factor type A domain protein (vWFA), which has been proven to be the most potent activator of SSA/Ro60-reactive T cells, consequently, inducing robust IL-2 production. In addition, recombinant vWFA protein and whole *E. coli* expressing vWFA are both capable of initiating the activation of Ro60-reactive T cells (38).

Besides, the highly-conserved microbial proteins of *Staphylococcus aureus* and *E. coli* can induce patients with SS to develop corresponding antibodies, and as a consequence of molecular mimicry, these antibodies cross-react against mitochondrial self-antigens, which may account for their fatigue symptoms (39).

These observations affirmed the concept of molecular mimicry between microbiota and the pathology of SS. Target genera that participate in this pathway still need to be clarified for various genera of the *Enterobacteriaceae* possessed proteins that have high amino-acid sequence similarities with OmpA and vWFA.

### Metabolite Changes

Short-chain fatty acids (SCFAs) are one of the important metabolites of intestinal microorganisms and are mainly composed of acetic acid, propionic acid, and butyric acid. As signaling molecules, SCFAs play a key role in regulating host metabolism, immune system and cell proliferation (40, 41). In systemic autoimmune diseases, several SCFA-producing bacteria, including *Lachnoclostridium*, *Lachnospira*, and *Sutterella* were reduced. Of note, these bacteria played a highly pro-regulatory, tolerogenic role in immune functions (42). In SS, butyrate is the most commonly mentioned bacterial metabolite. Several studies have found butyrate-producing bacteria (including *Faecalibacterium prausnitzii*, *Bacteroides fragilis*, *Lachnoclostridium*, *Roseburia*, *Lachnospira*, and *Ruminococcus*) were significantly decreased in SS (13, 15–17, 28). As a vital microbiota-derived metabolite, butyrate provides energy for colonic epithelial cells and promotes intestinal barrier functions. Moreover, recent research observed that butyrate-producing bacteria, *Bacteroides* spp. and the *Clostridia* clusters XIVa and IV were important for maintaining the Treg/Th17 balance. To be noted, the differentiation of Treg cells is not only promoted by butyrate but also by the immunomodulatory molecule polysaccharide A derived from the *Bacteroides fragilis* (43–45). Interrupting the Treg/Th17 balance will lose the protection of the mucosa barrier from colonization by pathogenic microorganisms (15). Actually, butyrate could both affect the balances in T cells and regulate the frequency of IL-10 and/or IL-17 producing B cells by regulating circadian-clock-related genes to perform the anti-inflammation function (46).

In other respects, butyrate could elevate the flow rate of salivation and the relief of salivary gland inflammation (46). All these clues suggest that the reduced SCFAs or butyrate-producing bacteria might affect the permeability of the mucosa barrier, the frequency or function of immune cells, and even secretion of the salivary gland, so as to be involved in the pathogenesis of SS.

### Epithelial Tolerance Breakdown

Breakdown of epithelial tolerance exists in SS but the mechanism has not yet been clarified. Some studies observed that salivary gland epithelial cells (SGECs) were expressed with immune checkpoint molecules that may have a potential role in the immune response (47) and bacteria involvement may be an important reason for this phenomenon. *H. parainfluenzae* was considered to act as an immunomodulatory commensal bacterium in SS, as its reduction may result in decrease of the PD-L1 on SGECs, whose inhibitory control on the proliferation of CD4+T may be loosened according to the observation found in the pretreatment of A253 cells (from human submandibular gland squamous cell carcinoma) with *H. parainfluenzae* (29).

Interestingly, Jehan et al. reported the involved salivary gland of SS was infected by bacteria including *Prevotella melaninogenica* (25). *Prevotella* is an SS-associated oral bacteria that could upregulate MHC molecules and CD80 in human submandibular gland tumor cells, which may initiate IFN pathway-related inflammation (13). Interestingly, the significantly higher abundance of *Prevotella* was found in a family that contains key enzymes for mucin degradation that could also affect the colonic mucus barrier (48). Other than reducing the supply of energy for epithelial cells, disrupting the immunomodulatory function of epithelial cells and interfering with the mucus barrier that protects the epithelial cells are the two other possible ways for the microbiota in SS to achieve the breaking down goal.

Besides molecular mimicry, metabolite changes, and epithelial tolerance breakdown, some correlation between bacterial alteration and pathological changes was observed but the causality needs further exploration. For example, in TSP-1-/- mice, *Staphylococcus aureus* and *coagulase-negative staphylococci* sp. *species* were significantly increased and associated with increased neutrophil infiltration into the conjunctiva (49). Importantly, the three links we presumed were not absolutely independent but might cross with each other, which makes the exploration of the causality between microbiota and SS more complicated.

## Gut-ocular and gut-oral axis

Exploring the clinical relationship/pathogenesis and the microbiome changes among the microbiota from gut, ocular, and oral, also called gut-ocular-oral axis, helps illustrate the role that bacteria plays in the development of SS. Mahira et al. observed that germ-free CD25 knockout mice have worsened involvement of gut, ocular, and oral. Also, the levels of pro-inflammation factors were increased and the percentage of CD4+IFN-γ+ cells was greater in this germ-free model. Noteworthy, fecal matter from normal mice being transplanted into CD25 knockout mice could help reverse the pathological changes in the gut, ocular, and oral areas (50). Therefore, the gut-ocular-oral axis might exist in SS.

Regarding the ocular-gut axis, several studies have shown that intestinal dysbiosis also has a connection with the severity of ocular mucosal diseases in SS. Moon et al. reported that the relative abundance of *Bacteroidetes*, *Actinobacteria*, and *Bifidobacterium* in the SS gut was significantly associated with dry eye symptoms (13). Animal models, pretreated with desiccating stress and antibiotics, were found to have extreme changes in gut microbiota, which was partly counting on the *Proteobacteria* found to be associated with the more severe ocular phenotype (17). These findings suggest that intestinal dysbiosis has a significant clinical association with the severity of dry eye. It is speculated that dysbiosis of the gut microbiota might aggravate germinal center loss in dry eyes by reducing the frequency of resident NK/NKT cells and changing their cytokine profile, leading to suppressed Th2 tone (17).

Worth mention, buccal epithelial cells inhabited by intracellular bacteria can be an important reservoir. The intracellular bacteria may be transmitted to the gut through the shedding process of buccal epithelial cells (51, 52). In SS, the relative abundance of *Actinomyces* and *Lactobacillus* in oral samples was associated with their abundance in stool samples (12). Moreover, we have described above that several studies have suggested the cross-reactivity of commensal oral and gut bacteria with SSA/Ro60 is of great importance in the pathogenesis of SS (36, 38). Therefore, it is plausible that there is a link between oral and gut bacteria but the related mechanism is still unknown.

It has been found that *Firmicutes*, *Actinobacteria*, *Proteobacteria*, and *Bacteroidetes phyla* are dominant in conjunctival samples from patients with SS and controls (17). However, as yet, no significant difference has been found in the diversity and composition of the ocular surface microbiome between SS and controls. Also, research on the ocular-oral axis is limited and no related result was observed.

## Hints for treatment

In recent years, targeted manipulation of the microbiome to restore immunity and improve disease outcomes has drawn close attention from researchers. Regulating the abnormal diversity of the microbiome was the main direction for SS therapy. Several studies have applied cohousing therapy, fecal transplant, and probiotic therapy among SS animal models. For example, Lee et al. found that cohousing young Nfkbiz+/+ with Nfkbiz−/− mice synchronized their oral microbiome toward that of young Nfkbiz+/+ mice, preventing dysbiosis of Nfkbiz−/− mice (28). Further, Zaheer et al. showed that fecal bacteria transplantation decreased the generation of pathogenic CD4+IFN-γ+ cells and reversed the spontaneous dry eye phenotype of germ-free CD25KO mice (50). Similarly, another study of an SS animal model showed that ocular surface disease was alleviated by probiotic therapy with 5 ingredients of *Lactobacillus reuteri*, *Streptococcus thermophilus*, *Bifidobacterium bifidum*, *Lactobacillus casei, and Lactobacillus acidophilus* (53). Future clinical trials performed in SS patients will be needed to confirm these exciting results.

Though the effect of therapeutic strategies involving the microbiome was not valid, the shift of microbiota during traditional treatment helps enrich the cognition of these therapies. Lu et al. demonstrated that treating SS patients with total glucoside of paeony (TGP) improved the diversity and the composition of the intestinal microbiome, including increasing the abundance of *Firmicutes*, thereby inhibiting the pathogenic role of *Bacteroides* (11). Moreover, TGP combined with hydroxychloroquine promoted the thriving of several commensal bacteria (*Lactobacillus*, *Incertae*, and *Desulfovibrio*) and limited the expansion of dominant pathogenic bacteria (*Bacteroides* and *Alloprevotella*) (11). To a certain extent, supervision of the microbial diversity and the composition of microbiota may not only help explain the pharmacological mechanism of drugs but also be a potential marker to evaluate the effectiveness of treatment.

## Summary

Microbiota of SS has been a popular issue in recent years. Generally, studies have shown that the SS microbiome is mainly characterized by reduced gut and oral diversity. In addition, a negative correlation was observed between the diversity and clinical symptoms. Based on current research, microbiota alteration may play their pathological role *via* at least three pathways: molecular mimicry, metabolite changes, and epithelial tolerance breakdown, which ultimately result in a dysregulated immune system and abnormal clinical symptoms such as dry eye and dry mouth. Causality studies are direly needed in this field, not only for the explanation of disease pathogenesis but also for the application of microbiota changes in clinical decision-making.

## Author Contributions

YF supervised the work. CD and QX collected the data and wrote the manuscript. CD was also responsible for the outline and modification of this review. All authors have read and approved the submitted version.

## Funding

This work was supported by the National Natural Science Foundation of China (grant numbers 81971545, 82172343) and the CAMS Innovation Fund for Medical Sciences (CIFMS, 2020-I2M-C & T-A-002).

## Conflict of Interest

The authors declare that the research was conducted in the absence of any commercial or financial relationships that could be construed as a potential conflict of interest.

## Publisher’s Note

All claims expressed in this article are solely those of the authors and do not necessarily represent those of their affiliated organizations, or those of the publisher, the editors and the reviewers. Any product that may be evaluated in this article, or claim that may be made by its manufacturer, is not guaranteed or endorsed by the publisher.
